# Connection between Cardiac Fibrosis Biomarkers and Echocardiography Parameters in Advanced Chronic Kidney Disease Patients

**DOI:** 10.3390/jcm12083003

**Published:** 2023-04-20

**Authors:** Carina Ureche, Gianina Dodi, Alexandra Covic, Alina Nedelcu, Simona R. Volovăț, Radu A. Sascău, Cristian Stătescu, Adrian Covic

**Affiliations:** 1 Department of Internal Medicine, Faculty of Medicine, Grigore T. Popa University of Medicine and Pharmacy of Iasi, 16 University Street, 700115 Iasi, Romania; carina.ureche@yahoo.com (C.U.);; 2 Prof. Dr. George I.M. Georgescu Institute of Cardiovascular Diseases, 700503 Iasi, Romania; 3 Biomedical Sciences Department, Faculty of Medical Bioengineering and Advanced Research and Development Center for Experimental Medicine, Grigore T. Popa University of Medicine and Pharmacy of Iasi, 9-13 Kogalniceanu Street, 700454 Iasi, Romania; 4 Department of Medical Oncology, Grigore T. Popa University of Medicine and Pharmacy of Iasi, 16 University Street, 700115 Iasi, Romania; 5 Nephrology Clinic, Dialysis, Renal Transplant Center, Dr. C.I. Parhon University Hospital, 700503 Iasi, Romania

**Keywords:** chronic kidney disease, global longitudinal strain, procollagen type I carboxy-terminal propeptide (PICP), procollagen type III N-terminal peptide (P3NP), galectin-3, myocardial fibrosis

## Abstract

Background: Myocardial fibrosis represents a mainstay pathway in the pathophysiology of uremic cardiomyopathy. This process leads to structural and functional changes in the heart, which can be detected by echocardiography. The purpose of our study was to determine the association between four echocardiographic parameters (ejection fraction (EF), global longitudinal strain (GLS), mean E/e’ ratio, and left atrial volume indexed) and biomarkers associated with cardiac fibrosis, such as procollagen type I carboxy-terminal propeptide (PICP), procollagen type III N-terminal peptide (P3NP), and galectin-3 (Gal-3) in patients with end-stage renal disease (ESRD). Methods: 140 patients with ESRD were enrolled and investigated by echocardiography and the serum levels of the aforementioned biomarkers were determined at baseline. Results: The mean EF was 53.63 ± 8%, the mean GLS was −10.2 ± 5.3%, the mean E/e’ ratio was 9.8 ± 4.3, and the mean left atrial volume indexed (LAVI) was 45.8 ± 14.2 mL/m^2^. The average levels for PICP, P3NP, and Gal-3 were 457.2 ± 240 µg/L, 242 ± 199.9 µg/L, and 10.7 ± 3.7 ng/mL, respectively. In regression analysis, PICP was strongly associated with all four echocardiographic parameters (EF: *p* = 0.0002, R^2^ = 0.69; GLS: *p* = 0.00001, R^2^ = 0.81; mean E/e’: *p* = 0.00002; R^2^ = 0.89; LAVI: *p* = 0.003; R^2^ = 0.73). P3NP and Gal-3 were only associated with the EF (*p* = 0.01, R^2^ = 0.31 and *p* = 0.02; R^2^ = 0.35, respectively). Conclusion: Our study evidenced that PICP, a collagen-derived biomarker, is associated with important echocardiography parameters, suggesting that it can serve as an indicator of the presence of subclinical systolic and diastolic dysfunction in patients with advanced CKD.

## 1. Introduction

Patients with chronic kidney disease (CKD) present an elevated cardiovascular risk, being considered the leading cause of death in this high-risk population [1]. When these two entities coexist, the affected individuals exhibit more symptoms, frequent hospitalizations, and higher rates of morbidity [2].

The pathophysiological image of cardiovascular disease (CVD) in patients with ESRD includes traditional associated factors such as sympathetic overactivity, diabetes, dyslipidemia, and smoking, and the unconventional ones represented by inflammation, vascular calcifications, increased arterial stiffness, and autonomic instability [1,3]. These factors lead to the development of changes in myocardium structure and function characterized by left ventricular hypertrophy (LVH), accompanied by coronary microvascular disease, calcifications, and myocardial fibrosis, contributing to the high burden of heart failure with preserved EF [4]. Patients with CKD not only show an increased risk of sudden cardiac death but also have clear differences from the general population in terms of the pathophysiology and cause of sudden cardiac death.

Cardiac fibrosis, described by the increased collagen type I deposition and cardiac fibroblast activation and differentiation into myofibroblasts, is a common pathophysiologic escort of most heart diseases such as myocardial infarction, different types of cardiomyopathies, and hypertensive heart disease [5]. These cardiac events, lead to cardiac dysfunction decline since excessive extracellular matrix (ECM) deposition results in poor tissue compliance and hardening of myocardium [6]. Cardiac fibrosis also drives chamber dilation, cardiomyocyte hypertrophy, apoptosis, and congestive heart failure; thus, detection of potential diagnostic targets is an important goal for improving the morbidity and mortality of CKD patients [7].

Myocardial biopsy is the gold-standard method for quantifying myocardial fibrosis, defined as an excess of collagen fibers within the myocardium. In advanced CKD, an excessive accumulation of collagen type I takes place as a result of an accelerated synthesis and decreased degradation [1]. Since myocardial biopsy is an invasive method, not available in many centers, the idea of noninvasive quantification of myocardial fibrosis in ESRD is challenging.

Echocardiography is a useful tool in the detection of the structural and functional cardiac disease changes and may indicate the development of myocardial fibrosis in persons with advanced CKD. Conventional echocardiography is a helpful instrument in everyday practice, but new methods such as two-dimensional speckle tracking echocardiography were developed with the goal of detecting early subclinical abnormalities. The left ventricular GLS is a parameter derived from this technique, with great prognostic value for patients with CKD, confirmed in various studies so far [8,9,10,11].

Clinically available cardiac biomarkers, such as PICP, procollagen type I N- terminal propeptide (PINP), P3NP, C- terminal telopeptide of collagen type I (CITP), matrix metalloproteinases (MMPs), tissue inhibitors of metalloproteinase (TIMPs), Transforming Growth Factor-β (TGF-β), specific E3 ubiquitin protein ligase 1, connective tissue growth factor (CTGF), corin, mesenchymal cell products, and inflammatory factors, recommended for noninvasive assessment of myocardial fibrosis, were investigated extensively [12] and were found to be associated with the prognosis of various cardiovascular diseases in general population. Moreover, Gal-3 serum levels, a soluble biomarker for cardiac fibrosis, are associated with cardiac tissue remodeling in animal study, but further studies are needed to investigate the hypothesis in human patients [13]. Thus, collagen-derived biomarkers, namely PICP, P3NP, and Gal-3, that are implicated in fibrosis generating pathways, were shown to be associated with cardiac fibrosis [14,15]. Moreover, there are recent data proving that collagen-derived biomarkers are useful in several cardiac diseases and can help with the selection of patients who can benefit from anti-fibrotic interventions [16,17,18].

It is well-known that cardiac fibrosis involves multiple cells and molecular pathways with molecular markers used for the identification of mast cells, endothelial cells, macrophages/monocytes, cardiomyocytes, and cardiac fibroblasts that have an important role in the fibrotic response. Among these biomarkers, fibrogenic growth factors such as platelet-derived growth factor (PDGF) and TGF-β, inflammatory cytokines (TNF-α and IL-6), neurohumoral pathways (the renin–angiotensin–aldosterone system, GPCR/adrenergic signaling, endothelin-1), and exosomes may deliver a novel promising strategy for cardiac fibrosis diagnosis [6].

In the same trend, the current pattern of kidney disease detection benefits from the modern advancement in molecular analysis and identifies biomarkers for evaluating and characterizing kidney diseases; therefore, the most promising biomarkers with a strong foundation in the pathophysiology of kidney disease were found to be neutrophil gelatinase-associated lipocalin (NGAL), kidney injury molecule-1 (KIM-1), TIMP-2, α1-Microglobulin, liver-type fatty acid-binding protein (L-FABP), uromodulin (UMOD), interleukin-18 (IL-18), TNF-α and IL-1β, P3NP, and epidermal growth factor (EGF) [19]. As expected, the existence of kidney diseases can lead to a problematic analysis of cardiac biomarkers vital in accurate diagnosis and prompt management of CVD; therefore, associations between cardiac biomarkers and clinical outcomes are necessary. A related study investigated in 2020 the associations between established biomarkers (NT-proBNP and troponin T (hsTnT)) and novel biomarkers (growth differentiation factor 15 (GDF-15), Gal-3, and soluble ST-2), using echocardiographic measurements in persons with CKD, and their results showed that the novel GDF-15 along with NT-proBNP and hsTnT, were associated with echocardiographic measurements of subclinical cardiovascular disease [20].

In this context, the purpose of our study was to determine the association between four echocardiographic parameters (EF, GLS, mean E/e’ ratio, and LAVI) and biomarkers associated with cardiac fibrosis, such as PICP, P3NP, and Gal-3, in patients with end-stage renal disease. To the best of our knowledge this is the first study that evaluates and compares associations of PICP, P3NP, and Gal-3 biomarkers with echocardiographic measurements in patients with ESRD. Preliminary data from this study were previously reported at the European Society of Cardiology Congress in 2022 [21].

## 2. Materials and Methods

### 2.1. Study Population

Our study took place between October 2019 and October 2022 in the Nephrology Department of the Clinical Hospital Dr. C. I. Parhon from Iași, Romania.

**Figure 1:**
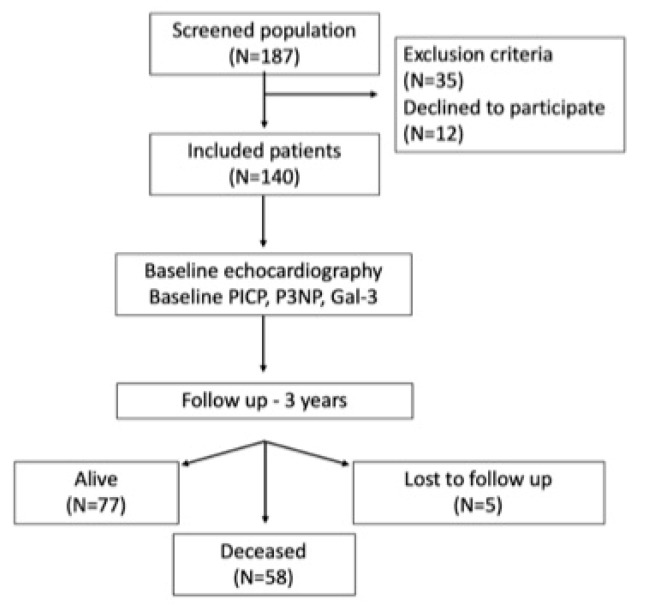
Study design. PICP: procollagen type I carboxy-terminal propeptide; P3NP: procollagen type III N-terminal peptide; Gal-3: galectin-3.

We included 140 patients with ESRD, not yet on dialysis, who had an estimated glomerular filtration rate (GFR) of ≤15 mL/min/1.73 m^2^, were asymptomatic, hemodynamically stable, and in sinus rhythm. The exclusion criteria included a poor acoustic window, atrial fibrillation, the presence of a cardiac pacemaker, an indication for emergency hemodialysis, NYHA class IV, severe pulmonary hypertension, active malignancy, or other pathologies with a life expectancy of less than one year. The study design is depicted in Figure 1.

### 2.2. Study Design

This was a cross-sectional cohort study in adult patients, for which the approval of the hospital’s ethics committee was obtained prior to the study. For investigating the association with mortality, an extension cohort study was added, with a follow-up period of three years. The primary endpoint at three years was all-cause mortality. Secondary endpoints included cardiovascular death, starting of dialysis, or kidney transplantation. Follow-up was carried out either by telephone-based approach, or by accessing the electronic health records of the Clinical Hospital Dr. C. I. Parhon in Iași.

All study participants provided written informed consent, and ethical committee board at the participating site approved the study protocol. This research was conducted in full agreement with the Declaration of Helsinki.

The patients who met the inclusion criteria were examined clinically and with echocardiography. All participants underwent venous blood sampling immediately prior to echo study. Bloods samples were spun and frozen at −80 °C and analyzed subsequently using standardized commercially available kits.

There were no interferences to be corrected related to the medication or hydration of the patients during the study. The presence or absence of a preemptively performed arterio-venous fistula was not considered exclusion criteria.

### 2.3. Transthoracic Echocardiography

All echocardiographic evaluations were performed using the Philips CX50 ultrasound system (Andover, MA, USA), with the help of QLAB 7.1 software (Andover, MA, USA). In order to minimize the variability, the ultrasound analysis was performed by the same observer according to the recommendations of the American Society of Echocardiography and the European Association of Cardiovascular Imaging [22]. Later, to ensure an unbiased outcome, one other investigator re-examined and interpreted the offline images.

All patients benefited from a comprehensive echocardiographic evaluation of both the cardiac anatomy (diameters, volumes, and mass) and of the functionality, the systolic and diastolic function of the left and right ventricles, atrial function, valvular function, and pulmonary artery pressure.

In this study, we chose to focus on the parameters of the systolic function of the left ventricle. First of all, the ejection fraction of the left ventricle was calculated by the biplane Simpson method, using 2D echocardiography in end diastole and end systole, using two standard views, apical four chambers, and apical two chambers. Cut-off values of 52% and 54% were used to define a normal ejection fraction in men and women, respectively [22]. Using the M-mode measurement of the mitral annulus movement, the mitral annular plane systolic excursion (MAPSE) value, an indicator of the longitudinal function of the left ventricle, was estimated, with a normal value of over 11 mm.

In addition, the global longitudinal strain of the left ventricle was calculated after obtaining three standard views (apical four chambers, apical two chambers, and apical three chambers), with the help of the QLAB 7.1 software (Andover, MA, USA). Cine loops with good image quality and high frame rate were used. After manually tracing three points of interest in end diastole, the software automatically traced the endocardial contour, which was corrected as needed. The region of interest (ROI) was automatically estimated and manually adjusted to be adapted to the left ventricular wall thickness. Subsequently, the global longitudinal strain was calculated, with the generation of the bullseye plot. For this study, we used the mean value reported by the two observers. A normal GLS value was considered to be >−18.9%, adapted to the used vendor [22].

### 2.4. Determination of Serum Biomarkers

Enzyme linked immunosorbent assay (ELISA) was used to measure the serum level of PICP, P3NP, and Gal-3. Peripheral blood samples were collected into serum separator tubes during the clinical examination and samples were allowed to clot for 2 h at room temperature. The serum tubes marked with a specific ID were separated by centrifugation (10 min. at 4500 rpm) and stored in aliquots of 2 mL Eppendorf tubes at −80 °C for subsequent ELISA.

Aliquots of serum samples were thawed naturally at room temperature for each assay. The level of PICP (cat. no. MBS026891), P3NP (cat. no. MBS045955), and Gal-3 (cat. no. MBS722196), purchased from MyBioSource Inc., San Diego, CA, USA, were used according to the manufacturer’s recommendations. Briefly, 50 or 100 µL of standards, blanks, and samples were added into the marked ID wells, followed by 100 or 50 µL of HRP-conjugate reagent and incubated at 37 °C for 1 h. The plate was then washed 4–5 times using a multi-channel pipette and 50 µL of substrate A and 50 µL of substrate B were added to all wells, incubated at 37 °C for 15–25 min., followed by 50 µL of stop solution addition to each well, and the optical density was determined at 450 nm using the Biochrom EZ Read 400 Microplate Reader (Biochrom, Cambridge, UK). The data were generated in electronic format with the help of the Galapagos Expert Software (Biochrom, Cambridge, UK). All measurements were performed in triplicate.

Afterward, the results were calculated using the standard curve constructed by plotting the average absorbance of standards against the known concentrations of standards (25–800 ng/mL for PICP, 3.12–100 ng/mL for P3NP and 2.5–50 ng/mL for Gal-3) using MyAssays online software. The levels of all biomarkers were expressed as nano-grams per millilitre of serum (ng/mL) and were used to calculate subsequent statistics.

### 2.5. Statistical Analysis

The statistical analysis was performed using the SPSS Statistics 25 software (IBM, Armonk, NY, USA). Non-normally distributed variables were expressed as median with interquartile range (IQR), and normally distributed variables as mean ± standard deviation (SD). To determine the correlations between the variables, the Pearson correlation coefficient was used. Stepwise multivariate regression analysis including all univariate associates (*p* < 0.05) was used to assess the independent associations between variables. The independent T test was used for comparing the means of two independent groups.

## 3. Results

### 3.1. Baseline Characteristics

A total of 187 patients were screened for inclusion in the study, 12 patients declined to participate, and 35 patients were excluded according to the exclusion criteria.

A total of 140 hypertensive patients with end-stage chronic kidney disease, not yet on dialysis were included (78 men and 62 women) and followed-up for 3 years. A total of 88% of the patients started hemodialysis and 12% underwent kidney transplantation. A total of 58 patients (41.4%) died during the follow-up period.

The data in Table 1 show the characteristics of the included patients.

The average age of the included group was 59 ± 15 years. A total of 25.7% of the included patients were obese (BMI ≥ 30 kg/m^2^), 16.4% were smokers (daily smoking), and 33.5% were diabetics (HbA1c ≥ 6.5%). All patients were hypertensive (SBP ≥ 140 mmHg and/or DBP ≥ 90 mmHg) and the majority had grade 3 hypertension (SBP ≥ 180 mmHg and/or DBP ≥ 110 mmHg) (92.2%). A total of 11.4% of patients had a history of acute myocardial infarction, and 5.7% had been revascularized, either by angioplasty or by coronary artery bypass grafting. The average value of hemoglobin was 9.72 ± 2 g/dL and that of uric acid was 7.51 ± 2 mg/dL.

### 3.2. Echocardiographic Parameters

In the analyzed cohort, the mean width of the inter ventricular septum and the posterior wall was 13.1 ± 1.8 mm and 13 ± 2.2 mm, respectively, indicating a high prevalence of LVH. The mean left ventricular end-diastolic diameter was 51 ± 5.8, with a mean left-ventricular end-diastolic volume of 137.1 ± 46.3 mL. When looking at the systolic parameters of the left heart, the mean EF determined by the Simpson biplane method was 53.63 ± 8%, with 52.1% of the patients in NYHA class I and 47.9% of patients in NYHA class II. In the analyzed population, GLS varied between −1% and −23%, with an average value of −10.2 ± 5.3%. The mean E/e’ was 9.8 ± 4.3, with 90.4% of the patients having at least grade I diastolic dysfunction. The mean left atrial volume indexed was 45.8 ± 14.2 mL/m^2^, indicating a dilation of the left atrium (cut-off value: 34 mL/m^2^).

When regarding the right part of the heart, we observed a mean right atrial volume of 52.7 ± 26.4 mL and a mean right ventricular fractional area change (RV FAC) of 42.3 ± 5.9%. The mean tricuspid annular plane systolic excursion (TAPSE) was 23.1 ± 3.8 mm, with a mean S’ velocity of 9.3 ± 2.6 cm/s. The mean inferior vena cava diameter was 17 ± 3.5 mm, indicating a normal volemic status (Table 2).

### 3.3. Serum Biomarkers

The average levels for PICP, P3NP and Gal-3 were 457.2 ± 240 µg/L, 242 ± 199.9 µg/L, and 10.7 ± 3.7 ng/mL, respectively.

### 3.4. Regression Analysis

We then focused on studying which of the three biomarkers were more likely to predict a decreased left ventricular systolic or diastolic function (decreased EF and decreased GLS; increased E/e’ and increased LAVI, respectively). For this, we performed regression analysis (Table 3 and Table 4).

**Figure 2:**
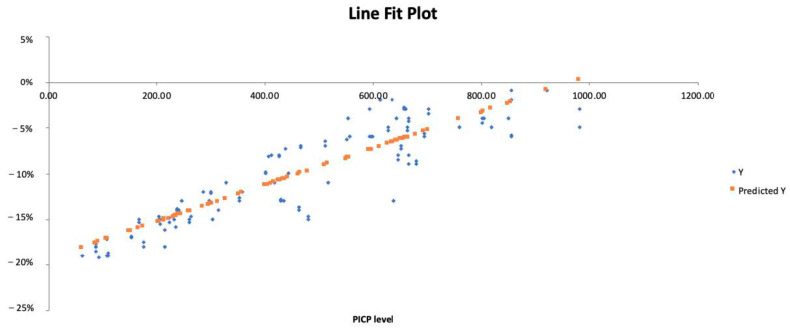
Regression analysis, line fit plot of GLS and PICP. Y = GLS.

In regression analysis, PICP was associated with the ejection fraction, GLS, mean E/e’ and LAVI (*p* = 0.0002, R^2^ = 0.69; *p* = 0.0001, R^2^ = 0.81; *p* = 0.00002, R^2^ = 0.89; *p* = 0.003, R^2^ = 0.73, respectively). The Line Fit Plot of PICP and GLS is depicted in Figure 2.

P3NP and Gal-3 levels were associated with the ejection fraction (*p* = 0.01, R^2^ = 0.31 and *p* = 0.02, R^2^ = 0.35, respectively), but the analysis for the other parameters did not reach statistical significance.

### 3.5. Subgroup Analysis by Diabetes and Smoking Status

In this cohort, 33.5% of the patients were diabetics. GLS was significantly higher in patients without diabetes mellitus (−11.4 ± 6%) compared with patients with diabetes (−9.3 ± 5%), *p* = 0.001. LAVI was significantly higher in patients with diabetes (49.18 ± 10.69 vs. 44.09 ± 15.44, *p* = 0.001). There were no significant differences for the EF and mean E/e’ between the two groups. PICP and Gal-3 levels were higher in patients with diabetes (475.54 ± 232.5 vs. 420.98 ± 253.04 µg/L, *p =* 0.003 and 11.25 ± 2.44 vs. 10.47 ± 4.2, *p* = 0.02, respectively). The values for P3NP did not reach statistical significance (240.37 ± 198.51 vs. 243 ± 201.7 µg/L, *p* = 0.09). After three years of follow-up, there were 31 deaths recorded in patients with diabetes (53.3%) and 27 deaths in the non-diabetic population (21.9%).

A total of 16.4% of the included patients were active smokers. The EF was significantly higher in the non-smoking group (58.68 ± 8.38 vs. 53.47 ± 6.69, *p* = 0.003). The mean GLS was higher in the non-smoking group, but the results did not reach statistical significance (−10.5 ± 5.4% vs. −9 ± 5%, *p* = 0.08). There were no significant differences in the mean E/e’ and LAVI between the two groups. There were significant differences in the PICP and Gal-3 levels between the smoking and non-smoking group (498.53 ± 226.51 vs. 449.1 ± 242.75 µg/L, *p =* 0.002 and 11.29 ± 2.33 vs. 10.62 ± 3.95, *p* = 0.01, respectively). The values for P3NP did not reach statistical significance (247.74 ± 118.21 vs. 241 ± 212.74 µg/L, *p* = 0.1). After three years of follow-up, there were 13 deaths in the smoking group (56.5%) and 45 deaths in the non-smoking group (38.4%).

Table 5 depicts the baseline characteristics of the alive and deceased groups at three years. The patients from the deceased group were significantly older, with a lower BMI, and diabetic. They had a lower ejection fraction, a lower global longitudinal strain, and higher levels of PICP, P3NP, and Gal-3.

## 4. Discussion

This paper aimed to investigate whether three biomarkers known to be indicators of organ fibrosis (PICP, P3NP, and Gal-3) are associated with echocardiographic parameters in patients with advanced CKD, bringing new evidence in the context of an understudied high-risk population.

In CKD, as an adaptive mechanism for the volume and pressure overload, the left ventricle responds with both hypertrophy and dilatation. In the context of LV hypertrophy, the interstitial space increases alongside an augmentation of the myocardial demands in oxygen, leading to myocardial fibrosis and accumulation of collagen, explaining the subclinical systolic dysfunction. In our study, as surrogates of the systolic function of the LV, we chose two parameters, LV ejection fraction, and global longitudinal strain. Despite a near normal mean ejection fraction of 53%, our patients presented low values of GLS, suggesting subclinical systolic dysfunction. Moreover, the mean GLS value in this cohort was −10.2 ± 5.3% (versus −19.4 ± 1.86% in the normal population), in line with the data published by Hensen et al. regarding this parameter in patients with CKD [8]. This finding is explained by the excellent ability of GLS to detect subclinical myocardial damage secondary to a high level of diffuse myocardial fibrosis, which was demonstrated by cardiac magnetic resonance studies. In contrast, EF can remain in the normal range for a large period of time, mostly because of the compensatory LVH [8]. The same group proved that a GLS below −10.6% was associated with all-cause mortality (HR 2.18, 95% CI 1.17 to 4.06, *p* = 0.014) [8,9,10].

Regarding the diastolic function of the left ventricle, we chose to focus on the mean E/e’ ratio and LAVI, with over 90% of the included patients presenting with diastolic dysfunction. It is important to point out that the mean E/e’ ratio in our cohort was 9.8 ± 4.3, suggesting normal filling pressures. This parameter represents an important tool for diagnosing diastolic heart failure and is correlated with mortality in CKD patients [27].

PICP, a product in the metabolism of collagen type 1, was proven useful for the diagnosis and prognosis of heart failure and is associated with GLS values in patients with diabetes [28,29]. Moreover, on endomyocardial biopsy, PICP indicates the presence of collagen type I, adding to the hypothesis that this molecule can serve as a biomarker of cardiac fibrosis [30]. In the HOMAGE trial, which included old patients at risk of heart failure, PICP, but not P3NP or Gal-3, was associated with structural and functional abnormalities. Moreover, in this study, spironolactone decreased PICP levels and alleviated the diastolic dysfunction, suggesting a potential benefit from this drug [31]. In both non-dialysis and dialysis CKD, higher levels of PICP are associated with an increased E/e’ ratio and LV filling pressure, suggesting a potential role of the collagen type 1 pathway in the increased stiffness which characterizes the uremic cardiomyopathy [32,33]. We previously reported data from this cohort, showing that PICP is an independent predictor of mortality in CKD patients [34]. Due to these findings we investigated the correlation with the echocardiographic parameters of the LV systolic and diastolic function. Our study confirmed, in regression analysis, that PICP levels are associated with the ejection fraction (*p* = 0.0002, R^2^= 0.69), GLS (*p* = 0.00001, R^2^ = 0.81), E/e’ ratio (*p* = 0.00002, R^2^ = 0.89), and LAVI (*p* = 0.003, R^2^ = 0.73). Taking all the data presented above into consideration, we can suggest that a combined multimodality imaging and biological approach can help estimate, in a non-invasive manner, the amount of cardiac fibrosis in CKD patients.

P3NP, a product in the metabolism of collagen type III, has prognostic value in heart failure, post-myocardial infarction can predict the incidence of AF and the recurrence after catheter ablation and the risk of ICD intervention [16]. Despite these promising studies, a recent report showed that the serum values of P3NP do not correlate with the incidence of CKD or heart failure [35]. This shifted the attention towards urinary levels of P3NP as a non-invasive ‘fibrotest’ in CKD [36]. Our study showed that patients with CKD exhibit higher baseline levels of serum P3NP compared with the general population, and these levels are associated with the ejection fraction (*p* = 0.01, R^2^ = 0.31). P3NP levels were not associated with the value of GLS, E/e’ ratio or LAVI.

Gal-3, a marker of inflammation is involved in aldosterone-mediated fibrosis, being an adjunctive novel biomarker that can be used in heart failure patients [37]. Along with soluble ST 2, it can be predictive of hospitalization and death in this population, and additive to natriuretic peptide levels concentration [38]. Considered a profibrotic agent in the kidneys, its elevated plasmatic concentrations can be detected before the development of chronic kidney disease [39]. The AURORA trial (a study designed for evaluation of Rosuvastatin in haemodialysis patients, in terms of survival and cardiovascular events, double-blind, randomized, multicentre study) investigated the association of two markers of fibrosis (Gal-3 and PICP) with cardiovascular outcomes: CV death and all-cause mortality. The results of the study revealed that there was a positive association between PICP and Gal-3 levels with CV death and all-cause mortality in this population [40]. In patients undergoing haemodialysis, a cut-off level of Gal-3 of >23.73 ng/mL was established as a predictor of mortality [41]. In CKD patients, Gal-3 was not significantly associated with left atrial or LV structure or function, as presented in cross-sectional analyses among 2101 participants with mild to moderate CKD in the Chronic Renal Insufficiency Cohort (CRIC) [20]. Even if in 2010, Gal-3 received a class II approval for risk prediction in heart failure, the association of Gal-3 with cardiovascular events is less evident, with significant and insignificant findings [42,43]. In our study, the mean Gal-3 levels were lower (10.7 ± 3.7 ng/mL) and were associated only with the ejection fraction (*p* = 0.02, R^2^ = 0.35).

The present study also evidences that smokers and patients with diabetes exhibit lower values of EF and GLS and higher values of PICP and Gal-3. This is in line with the literature data pointing towards a profibrotic status in these patients [44,45,46].

The limitations of this research include its design, which is not a prospective and randomized one, with no control group, followed by the unfeasibility to determine causality since it is an observational study.

## 5. Conclusions

In conclusion, this study evaluated the association between PICP, P3NP, and Gal-3 biomarkers and echocardiographic parameters in patients with end-stage kidney disease. Our research showed a strong association between PICP and all four echo parameters, as potential non-invasive indicators of myocardial fibrosis that impacts both systolic and diastolic dysfunction. In this cohort, P3NP and Gal-3 were only associated with the ejection fraction.

## Figures and Tables

**Table 1:** Baseline characteristics of the included patients.

Parameter (N = 140)	Value
Age (Average ± SD)	59 ± 15
Sex (Number, %)	62 F (44.3%), 78 M (55.7%)
Egfr (Average ± SD) (mL/min/1.73 m^2^)	8.7 ± 3.3
Creatinine (Average ± SD) (mg/dL, umol/L)	6.6 ± 2.4 (583.57 ± 212.21)
Mean duration of the renal disease (months)	43.2 ± 14.5
BMI (Average ± SD) (kg/m^2^)	27.04 ± 5
Obesity (Number, %)	36 (25.7%)
Smoking (Number, %)	23 (16.4%)
HTN grade (Number, %)	11 Grade 2 (7.8%), 129 Grade 3 (92.2%)
Systolic blood pressure (Average ± SD) (mmHg)	140 ± 15
Diastolic blood pressure (Average ± SD) (mmHg)	76 ± 5
Heart rate (Average ± SD) (beats/minute)	73 ± 4.8
NYHA class (Number, %)	Class I (52.1%), Class II (47.9%)
Diabetes mellitus (Number, %)	47 (33.5%)
History of MI (Number, %)	16 (11.4%)
CABG/PTCA (Number, %)	8 (5.7%)
Hemoglobin (Average ± SD) (g/dL)	9.72 ± 2
Uric acid (Average ± SD) (mg/dL)	7.51 ± 2
NT pro BNP (Average ± SD) (pg/mL)	250 ± 56
Mortality (Number, %)	58 (41.4%)

SD: standard deviation; Egfr: estimated glomerular filtration rate; BMI: body mass index; HTN: hypertension; NYHA: New York Heart Association; MI: myocardial infarction; CABG: coronary artery bypass grafting; PTCA: percutaneous transluminal coronary angioplasty; NT pro BNP: N-terminal pro B-type natriuretic peptide.

**Table 2:** Baseline echocardiographic data.

Parameter (N = 140)	Value (Mean ± SD)
Ventricular septum width (mm)	13.1 ± 1.8
Posterior wall width (mm)	13 ± 2.2
Left ventricular end-diastolic diameter (mm)	51 ± 5.8
Left ventricular end-systolic diameter (mm)	31.7 ± 7.4
Left ventricular end-diastolic volume (mL)	137.1 ± 46.3
Left ventricular end-systolic volume (mL)	63.6 ± 33.2
Ejection fraction (%)	53.63 ± 8
Left ventricular GLS (%)	−10.2 ± 5.3
Mean E/e’	9.8 ± 4.3
Left atrial volume indexed (mL/m^2^)	45.8 ± 14.2
Right atrial volume (mL)	52.7 ± 26.4
TAPSE (mm)	23.1 ± 3.8
S’ velocity (cm/s)	9.3 ± 2.6
Right ventricular fractional area change (%)	42.3 ± 5.9
Inferior vena cava (mm)	17 ± 3.5

SD: standard deviation; TAPSE: tricuspid annular plane systolic excursion.

**Table 3:** Values of GLS, PICP, P3NP, and Gal 3 in the analyzed population.

Parameter	Whole Sample (N = 140)	Normal Range (General Population)
GLS (%)	−10.2 ± 5.3 *	−19.4 ± 1.86 [23]
PICP (µg/L)	457.2 ± 240	50–350 [24]
P3NP (µg/L)	242 ± 199.9	1.2–4.2 [25]
Gal 3 (ng/mL)	10.7 ± 3.7	<17.8 ng/mL [26]

* Values are expressed in mean ± standard deviation. GLS: global longitudinal strain; PICP: procollagen type I carboxy-terminal propeptide; P3NP: procollagen type III N-terminal peptide; Gal-3: galectin-3.

**Table 4:** Regression analysis, GLS, EF, E/e’, LAVI, and serum levels of PICP, P3NP, and Gal-3.

	PICP (µg/L)	P3NP (µg/L)	Gal-3 (ng/mL)
Ejection fraction (%)	*p* = 0.0002	*p* = 0.01	*p* = 0.02
	R^2^ = 0.69	R^2^ = 0.31	R^2^ = 0.35
Global longitudinal strain (%)	*p* = 0.00001	*p* = 0.19	*p* = 0.3
	R^2^ = 0.81	R^2^ = 0.1	R^2^ = 0.08
Mean E/e’	*p* = 0.00002	*p* = 0.06	*p* = 0.2
	R^2^ = 0.89	R^2^ = 0.3	R^2^ = 0.1
LAVI (mL/m^2^)	*p* = 0.003	*p* = 0.42	*p* = 0.22
	R^2^ = 0.73	R^2^ = 0.04	R^2^ = 0.01

PICP: procollagen type I carboxy-terminal propeptide; P3NP: procollagen type III N-terminal peptide; Gal-3: galectin-3; LAVI: left atrial volume indexed.

**Table 5:** Baseline characteristics and comparison between the patients who survived and died at three years.

	Alive Group (*n* = 77)	Deceased Group (*n* = 58)	*p* Value *
Age (Average ± SD)	53.43 ± 15.9	67.47 ± 9.73	<0.0001
Sex (Number, %)	38 F (49.3%), 39 B (50.7%)	20 F (34.5%), 38 M (65.5%)	0.114
BMI (Average ± SD) (kg/m^2^)	27.2 ± 6.05	24.83 ± 4.37	0.02
Obesity (Number, %)	22 (28.5%)	14 (24.1%)	0.56
Smoking (Number, %)	11 (14.3%)	12 (20.6%)	0.361
NYHA class (Number)	46 Class I, 31 Class II	24 Class I, 34 Class II	0.038
Diabetes mellitus (Number, %)	15 (19.5%)	31 (53.4%)	<0.0001
Hb (Average ± SD) (g/dL)	9.96 ± 1.98	9.38 ± 1.55	0.06
Uric acid (Average ± SD) (mg/dL)	7.93 ± 1.7	6.92 ± 1.87	0.001
Ejection fraction (%)	54.48 ± 7.91	52.45 ± 8.32	0.146
Global longitudinal strain (%)	−10.86 ± 5.6	−9 ± 4.8	0.005
Mean E/e’	9.64 ± 4.15	10.13 ± 4.61	0.5
LAVI (ml/m^2^)	43.69 ± 12.71	48.79 ± 15.7	0.036
PICP (µg/L)	425.08 ± 258.8	502.66 ± 204.43	0.003
P3NP (µg/L)	240.69 ± 218.13	244.26 ± 172.9	0.0001
Gal 3 (ng/mL)	10.5 ± 4.02	11.07 ± 3.32	0.03

SD: standard deviation; BMI: body mass index; NYHA: New York Heart Association; * determined by the Fisher exact test for the categorical variables and by the independent T test for the continuous variables.

## Data Availability

Data supporting this study are not publicly available due to ethical restrictions. Please contact the corresponding author for further information.
